# Symptom Burden of Patients with Advanced Pancreas Cancer (APC): A Provincial Cancer Institute Observational Study

**DOI:** 10.3390/curroncol28040244

**Published:** 2021-07-22

**Authors:** Stephanie Lelond, Julie Ward, Pascal J. Lambert, Christina A. Kim

**Affiliations:** 1 CancerCare Manitoba Research Institute, CancerCare Manitoba, Winnipeg, MB R3E 0V9, Canada; ckim3@cancercare.mb.ca; 2 Max Rady Faculty of Health Sciences, College of Nursing, University of Manitoba, Winnipeg, MB R3T 2N2, Canada; 3 Rady Faculty of Health Sciences, University of Manitoba, Winnipeg, MB R3T 2N2, Canada; umward28@myumanitoba.ca; 4 Department of Epidemiology and Cancer Registry, CancerCare Manitoba Research Institute, Winnipeg, MB R3E 0V9, Canada; plambert@cancercare.mb.ca; 5 Section of Hematology/Oncology, Department of Internal Medicine, Rady Faculty of Health Sciences, University of Manitoba, Winnipeg, MB R3E 0W2, Canada

**Keywords:** symptom burden, pancreatic cancer, patient-reported outcomes, ESAS, prognosis

## Abstract

Patients with advanced pancreatic cancer (APC) experience many disease-related symptoms. ESAS-r measures the severity of 9 symptom domains and has been validated for use in the ambulatory oncology setting. We aimed to describe symptom burden at baseline for patients with APC treated with modern chemotherapy (CT), and to determine whether symptom burden at baseline is prognostic. Patients diagnosed with APC between 2012–2016, treated with ≥1 cycle of CT, who completed ≥1 ESAS-r were identified. Descriptive statistics were used to report symptom burden and common moderate-to-severe symptoms. A joint model was used to describe the trajectory of ESAS-r during follow-up while controlling for death. Multivariable Cox regression was used to identify independent predictors of death. Of 123 patients identified, the median age was 65 and 61% had metastatic disease. The median baseline ESAS-r total symptom distress score (TSDS) was 24. A total of 86% of patients had at least one symptom score of ≥4 at baseline, with the most common being: fatigue, nausea, anxiety, and shortness of breath. Median overall survival was 10.2 months. Baseline TSDS was not predictive for worse survival in the era of modern CT. Patients with APC have a high burden of cancer-associated symptoms and a high prevalence of moderate-to-severe symptoms. Early intervention has the potential to improve quality of life in this group of patients and should be investigated.

## 1. Introduction

Pancreatic cancer is the third leading cause of cancer death in Canada [1]. Most cases are diagnosed when disease is advanced and incurable [2]. Patients with advanced pancreatic cancer (APC) experience many disease-related symptoms, including pain, anorexia, weight loss, fatigue, nausea, diarrhea, depression, and anxiety [3,4,5,6]. Symptom burden increases closer to death [7,8]. 

The revised Edmonton Symptom Assessment System (ESAS-r) [9] is a patient-reported outcome (PRO) tool which assesses the intensity of nine symptoms, including pain, tiredness, drowsiness, nausea, appetite, dyspnea, anxiety, depression, and overall wellbeing. Each symptom is given a score on a scale of 0–10, with 0 defining the absence of a symptom and 10 defining the worst possible severity. A rating scale of 1–3 represents mild severity, 4–6 moderate severity, and 7–10 high severity [10,11]. The total symptom distress score (TSDS) is the sum of the 9 individual symptom scores with 90 being the highest possible total [12]. ESAS-r has been shown to be a reliable and valid tool in patients with advanced cancer in an outpatient oncology setting [9,13,14,15,16]. Since 2011–2012, ESAS-r has been completed by patients at each ambulatory visit at our provincial cancer center. Patients complete a paper copy, which clinical staff subsequently input into the electronic medical record. 

Over the last decade, combination chemotherapy regimens such as 5-fluorouracil, irinotecan and oxaliplatin (FOLFIRINOX) and *nab*-paclitaxel and gemcitabine (NG) have become the standard of care for APC, as they have been shown to improve survival, without a deterioration on quality of life (QOL), compared to single-agent gemcitabine [17,18,19]. The symptom burden soon after diagnosis in patients with APC who receive chemotherapy (CT) is not clearly defined. It is unknown whether higher baseline symptom burden is a poor prognostic factor for overall survival (OS) in patients with APC receiving modern CT. The aims of this study were:

1 To describe the symptom burden of patients with APC at baseline, using the ESAS-r.
2 To examine whether baseline ESAS-r is a prognostic marker for OS.

## 2. Materials and Methods

Patients ≥ 18 years of age and diagnosed with locally advanced unresectable or metastatic pancreatic cancer between 2012 to 2016 and treated with at least one cycle of CT in Manitoba were identified using the Manitoba Cancer Registry. Patients were included if at least one ESAS-r entry was present in the electronic medical record. All ESAS-r scores completed from baseline to death were extracted from the electronic medical record. The baseline ESAS-r measurement was completed within the 30 days preceding the start of CT. ESAS-r assessments with missing data were excluded. 

Baseline patient and treatment characteristics, including age, sex, date of diagnosis, clinical stage, Eastern Cooperative Oncology Group (ECOG) performance status, CT regimen received, date of progression, and date of death were extracted via the Manitoba Cancer Registry and retrospective chart review. 

The TSDS was calculated by adding up the sum of the scores for each of the 9 symptom domains. Box-percentile plots were used to demonstrate distributions. Symptoms were also categorized into a physical subset score (sum of pain, tiredness, drowsiness, nausea, lack of appetite and shortness of breath) and a psychological subset score (sum of depression and anxiety) for descriptive purposes. The correlations between TSDS and the physical and psychological subsets were assessed using Spearman correlation. 

Descriptive statistics were used to report baseline characteristics, symptoms at baseline, and the TSDS. TSDS according to primary tumor location and according to younger (<65) and older (≥65) age was explored. A joint model was used to describe the trajectory of ESAS-r during follow-up while controlling for death [20]. This is a model that joins a mixed model (predicting ESAS-r) and a survival model (predicting death). Splines were used to account for non-linear relationships and an interaction term between baseline TSDS and follow-up time was also included. To evaluate the impact of the time-varying status of TSDS during follow-up on death, a joint model was again used, which creates an endogenous variable through the mixed model to be applied in the survival model. R version 4.0.3 [21] was used to perform analyses using the R packages of JM [22] and rms [23]. OS was estimated using the Kaplan-Meier method. Multivariable Cox regression was used to identify independent predictors of death, including baseline TSDS.

## 3. Results

### 3.1. Patient Characteristics

A total of 123 patients diagnosed with APC from 2012–2016 who received at least one cycle of CT and completed at least one ESAS-r assessment were identified. Baseline characteristics can be seen in Table 1. The median age was 65 years (range 42 to 88), 52.8% were male, and 61% had metastatic disease. Most patients (82.1%) had an ECOG performance status of 0–1. The most common CT regimen received was 5-Fluorouracil, Irinotecan and Oxaliplatin (FOLFIRINOX) (69.1%), followed by *nab*-Paclitaxel plus Gemcitabine (NG) (22%). Only 11 (8.9%) patients received single-agent gemcitabine. Among the 123 patients, there were 1608 unique ESAS-r assessments. A total of 107 (87.0%) of patients completed ≥2 assessments, while 22.8% of patients had >21 completed assessments. In the cohort, 9.2% had at least 1 ESAS-r assessment with missing data, which were removed from analysis. 

### 3.2. Symptom Burden at Baseline

**Figure 1:**
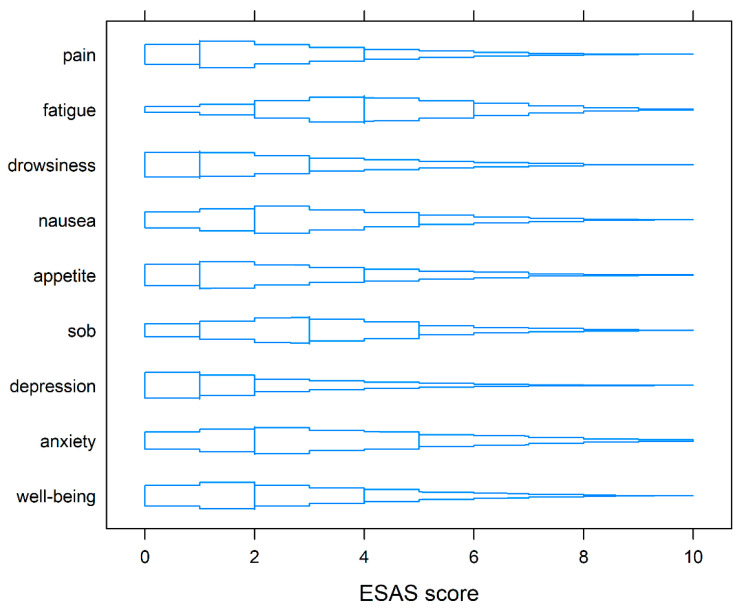
Box-percentile plots of symptoms on ESAS-r at baseline. Similar to boxplots, the median, 25th, and 75th percentiles are indicated with line segments across the box. Up to the median, the width indicates the percentile of that height. Beyond the median, the width indicates 100% minus the percentile.

At baseline, the median TSDS of the whole cohort was 24. ESAS-r TSDS in the 10th percentile and 90th percentile was 6.2 and 53, respectively. The median symptom scores at baseline for pain, tiredness, drowsiness, nausea, lack of appetite, shortness of breath, depression, anxiety, and wellbeing were 1, 4, 1, 2, 1, 3, 1, 2 and 2 respectively (Figure 1). 

Younger patients (under 65 years old) had a median TSDS of 29, whereas older patients (65 years and older) had a median TSDS of 21. At baseline, the median TSDS according to tumor location was 21.5 for a primary in the head/neck of the pancreas, 28 for the body of the pancreas, and 24.5 for the tail of the pancreas. 

At baseline, 106 (86.2%) patients reported at least one moderate-to-severe symptom (score ≥ 4). Ninety-five (77.2%) had at least one physical symptom scored at ≥4, while sixty-five (52.9%) had at least one psychological symptom scored at ≥4 at baseline. The most common symptoms with a reported score of ≥4 at baseline were tiredness (56.9%), anxiety (50.4%), shortness of breath (48.8%) and nausea (9.8%) (Table 2). 

Spearman correlations demonstrated high associations between baseline TSDS and physical symptom scores (rho = 0.94; *p* < 0.001) and psychological symptom scores (rho = 0.83; *p* < 0.001). Point-biserial correlations between TSDS and the subsets indicated moderate correlation. However, the results from multivariable models indicated little change when the subsets were included/excluded.

### 3.3. Symptom Burden over Time

**Figure 2:**
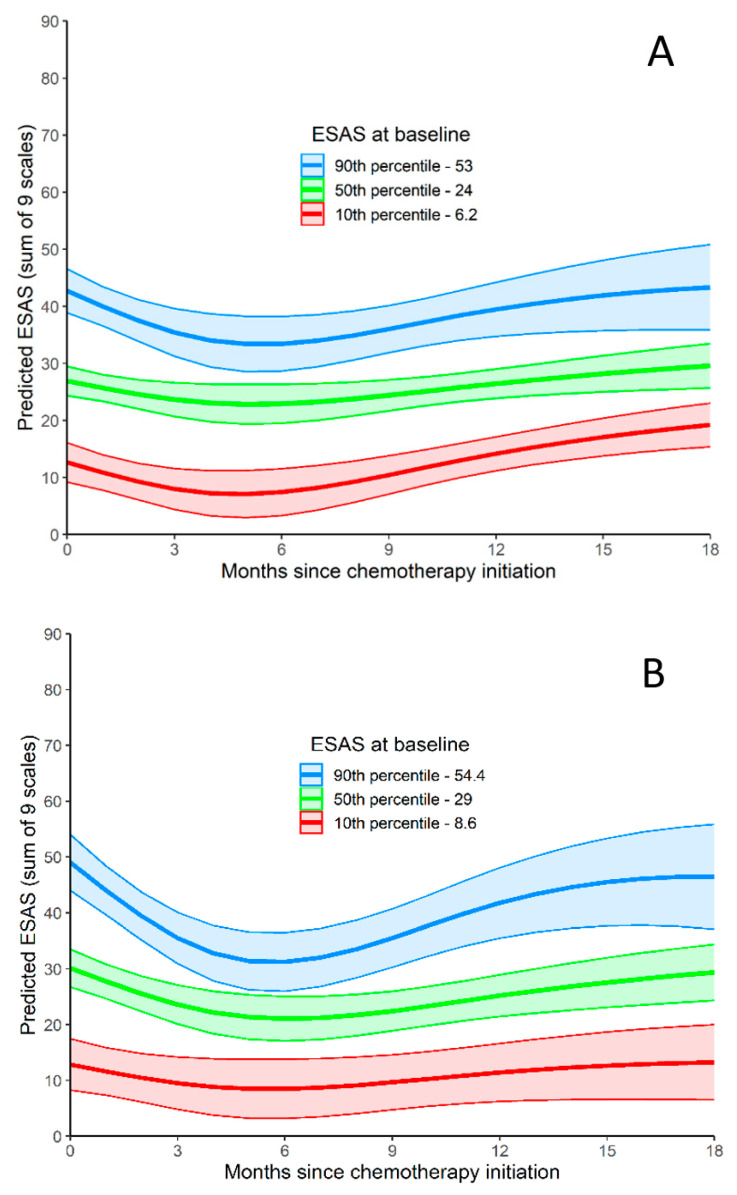

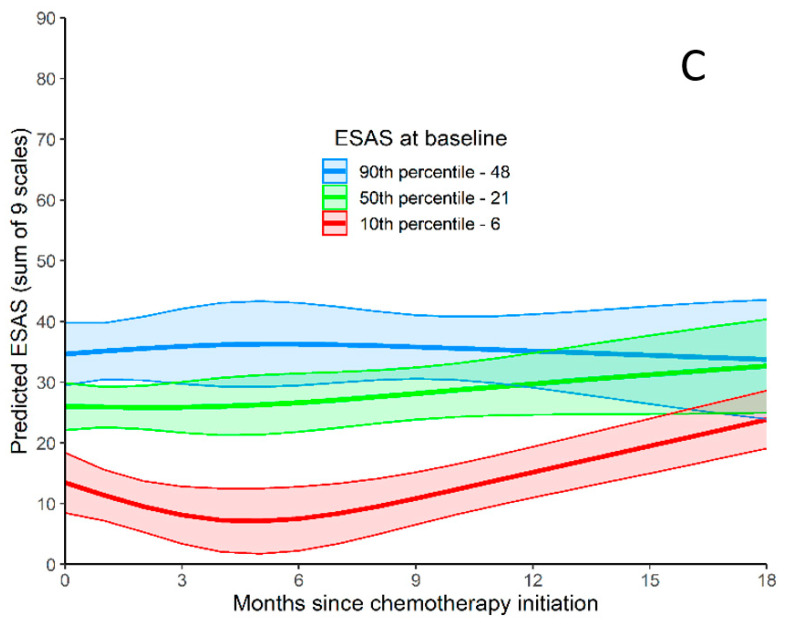
ESAS-r Total Symptom Distress Score. (**A**) ESAS-r Total Symptom Distress Score relative to months since chemotherapy initiation. Predicted outcome values are produced for three baseline ESAS-r values (values at the 10th, 50th, and 90th). (**B**) ESAS-r Total Symptom Distress Score for patients <65 relative to months since chemotherapy initiation. Predicted outcome values are produced for three baseline ESAS-r values (values at the 10th, 50th, and 90th). (**C**) ESAS-r Total Symptom Distress Score for patients >65 relative to months since chemotherapy initiation. Predicted outcome values are produced for three baseline ESAS-r values (values at the 10th, 50th, and 90th).

ESAS-r over time (0 to 18 months) is represented in Figure 2. Using a joint model (to predict TSDS values during follow-up while adjusting for death during follow-up), baseline TSDS was related to TSDS during follow-up. As seen graphically in Figure 2A, TSDS decreases for the first few months after initiating CT, but after 6 months, TSDS starts to increase. TSDS at 15 to 18 months is similar to TSDS at baseline. When disease progression was included as a time-varying predictor, it was not related to worsening TSDS (*p* = 0.750). When tumor location was included as a predictor of ESAS-r over time, patients with tumors in the head/neck and tail had higher ESAS-r scores than patients with tumors in the body of the pancreas (*p* = 0.032). 

This was found among younger patients, but not among older patients. When analyzed according to age, younger patients experienced a drop in ESAS-r during follow-up (Figure 2B), whereas older patients did not experience this same drop (Figure 2C). 

### 3.4. Survival Outcomes

The median progression free survival of the cohort was 6.7 months, and the median OS was 10.2 months. Multivariable Cox regression was used to identify independent predictors of death. The absence of metastatic disease and receipt of combination chemotherapy were associated with improved OS. TSDS at baseline and the presence of a physical or psychological symptom ≥ 4 were not prognostic for worse survival (Table 3). 

In a subgroup analysis of younger (<65) and older (≥65) patients, TSDS at baseline and the presence of a physical or psychological symptom ≥ 4 were not prognostic for worse survival.

However, a joint model that included time-varying TSDS indicated that higher TSDS during follow-up was related to a higher risk of death (Table 4). 

## 4. Discussion

Our study adds new information to a growing body of literature describing the symptoms of patients with cancer using a validated and widely used PRO tool. Previous studies have demonstrated that patients with a variety of cancer types experience moderate-to-severe symptoms in the period after cancer diagnosis [24,25,26], and while undergoing cancer treatment [27]. Because pancreatic cancer is a relatively rare diagnosis, it is often grouped together with other gastrointestinal cancers in large data sets. However, pancreatic cancer is particularly aggressive and fatal, and as such, understanding the constellation and severity of symptoms of this specific group of patients is beneficial. Although studies describing symptoms in APC using PROs are limited, the existing data are consistent with what is seen in our population, with the presence of moderate-to-severe symptoms at the time of diagnosis [28,29], while receiving treatment [6], and at the time of hospice enrolment [30].

The median baseline TSDS of our population was 24, which is similar to what has been reported in another group of patients with APC receiving CT [29], and higher than what has been described in many other cancer types [31,32]. For example, in a report of patients with metastatic renal cell carcinoma receiving systemic therapy, the median baseline TSDS was 16 [32], and in a cohort of patients diagnosed with a variety of cancer types, including breast, colorectal, lung, prostate, and hematologic cancers, the mean TSDS at baseline was 22.9 [31]. Most patients (86%) in our cohort had at least one moderate-to-severe physical symptom, and over half of patients had at least one moderate-to-severe psychological symptom at baseline. Comparatively, in metastatic lung, colorectal, prostate, and breast cancers, 42.3%, 26.7%, 24.5%, and 22.6%, of patients respectively reported moderate-to-severe physical symptoms, while 33.4%, 24,3%, 19.5% and 26.1% respectively reported moderate-to-severe psychological symptoms [25]. Similar to what has been reported in other cancer populations [6,24,26,28,29,30,33,34], fatigue/tiredness was a major symptom in our cohort. Other common moderate-to-severe symptoms in our population included anxiety, shortness of breath, and nausea.

There is limited data assessing the symptom burden of patients with APC receiving active CT treatment. In a phase 1 study of patients with locally advanced pancreatic cancer, receiving chemoradiation with capecitabine and bevacizumab, the most common moderate-to-severe symptoms using the MD Anderson Symptom Inventory were fatigue, loss of appetite, pain, distress, and drowsiness. Prior to starting treatment, 42% reported at least one moderate-to-severe symptom, and the presence of comorbid medical conditions was associated with symptom severity [28]. In a population-based retrospective review of ESAS-r records of patients with APC, receiving CT, moderate-to-severe symptoms included fatigue, lack of appetite and lack of wellbeing [29]. In a study prospectively describing symptoms of patients with APC, fatigue, loss of appetite, and impaired wellbeing were also prominent at baseline; however, only 6 of 51 included patients received CT [35]. In an integrative review describing symptoms of patients with APC, assessed with 9 different PROs, including ESAS-r, major symptoms included fatigue, lack of appetite, pain, gastrointestinal symptoms, anxiety, and depression. However, it is unclear how many patients included in this review received systemic therapy [6].

The lack of moderate-to-severe pain in our study may reflect the unique nature of our cohort, which was patients with APC who were functionally well enough to receive palliative intent modern CT. However, even in a group of patients with APC requiring hospice care [30], 28% of patients did not report pain. Others have hypothesized that tumor location and proximity to the celiac plexus may be responsible for differing reports of pain [29].

In an assessment of the symptom trajectory in our cohort, symptoms improved after starting CT, but then started to deteriorate after 5 to 6 months. In a study of patients with APC receiving chemoradiation, there was an improvement in pain but worsening fatigue, sleep, and nausea during treatment, and a significant improvement in overall moderate-to-severe symptoms after treatment [28]. Interestingly, the 5-to-6-month range in which the symptoms of our cohort began to deteriorate corresponds with the median progression free survival with modern CT regimens [18,19]. This is an intriguing observation; however, a detailed investigation of symptom trajectory is outside of the scope of this study.

In our cohort, both baseline TSDS and the presence of a moderate-to-severe symptom were not associated with shorter survival. In a study of patients with advanced renal cell carcinoma receiving palliative intent therapy, baseline ESAS-r was prognostic [32]. In another study of patients with advanced cancer that did not include APC, both TSDS and the presence of a moderate-to-severe physical symptom, were associated with inferior survival [25]. In a population-based study of patients with APC receiving CT, patients who had a higher TSDS had a worse OS [29]. However, it should be noted that 54% of patients in this study received single-agent gemcitabine as first-line chemotherapy, while less than 40% received FOLFIRINOX, and only 6% received NG.

The difference seen in our study may be more reflective of the impact of a modern-day CT approach, with agents which are known to improve not only OS, but also disease-associated symptoms and QOL [36,37]. It is plausible that in patients who receive combination CT, baseline TSDS is no longer prognostic because patients experience both symptom and survival improvement with CT. Our study shows that TSDS later in the disease trajectory is more telling in patients with APC receiving modern CT. Other studies have also shown that in patients with advanced cancer, symptoms worsen before death [7], and that higher TSDS is associated with a shorter time to death [8].

The impact of novel therapies on symptom burden in APC is also of interest. Clinical trials thus far have shown minimal impact of immunotherapy on outcomes in APC [38,39,40]. Studies assessing the role of immunotherapy in combination with chemotherapy have shown some promise [41,42,43]; however, biomarker analyses and results of prospective phase III trials are awaited. Immunotherapy has been shown to be beneficial in the treatment of mismatch repair deficient (dMMR) or microsatellite high (MSI-H) disease, which occurs rarely, in 1 to 2% of patients with pancreatic cancer [44]. In the phase II KEYNOTE-158 study of patients with non-colorectal dMMR or MSI-H cancers, among 22 patients with pancreatic cancer, 4 experienced a radiologic response to the anti-PD-1 monoclonal antibody, Pembrolizumab [45]. In an assessment of tumor mutational burden (TMB) in KEYNOTE-158 [46], patients with a high TMB also experienced an increased response to immunotherapy. Assessing symptom burden in the subset patients with APC treated with immunotherapy will be informative in future studies.

There are important limitations to this study. Many patients with APC are not candidates for CT for a variety of reasons, including age, comorbidities, and poor functional status. It is possible that patients who are not candidates for CT have a different symptom profile. As such, our results may not be representative of all patients with APC. Due to the retrospective nature of our study, we could not control for all known prognostic variables, but included clinically relevant variables in our analysis, including clinical stage, ECOG, age and CT type received. The retrospective design also limits the ability to state why patients received the treatment that they did. This is particularly pertinent to the 9 patients who received single-agent gemcitabine; however, during the beginning of the time period of our study, gemcitabine would have still been a relatively common CT regimen for APC, and this regimen can still be considered in patients who wish for less toxicity, patients who are elderly, or those with a poor functional status. Our sample size is small, as it was limited to those who received CT and completed ESAS-r. Inconsistent results by subgroups such as age and tumor location may be due to limited power in the subgroup analyses. ESAS-r completion is voluntary at all outpatient visits at our institution. It is possible that some patients declined to complete the ESAS-r, and symptoms of patients admitted to hospital would not have been captured. ESAS-r began to be routinely collected at our institution in 2012. It is completed by patients on paper and then transcribed by clinical staff into the electronic medical record. It is possible that some PROs were missed in the transcription process. These factors limit the generalizability of our results to a broader population of patients with APC. 

Our study confirms that in a select group of patients with APC, there is a high burden of symptoms at baseline, a high prevalence of moderate-to-severe symptoms, along with a poor prognosis. Although ESAS-r is a tool that measures symptom burden and not QOL, our findings suggest that investigating QOL in patients with APC is important. Poor QOL has been reported in pancreatic cancer [47,48,49], and studies have reported an association between poor QOL and shorter survival [47,50,51,52]. As APC is a lethal disease, with a high symptom burden, it is critical to explore how QOL can be improved for this group of patients. Early interventions, such as pain and symptom management or automatic palliative care referral, could be considered [9,53,54,55,56]. Using baseline symptoms and symptom trajectory as opportunities for intervention may result in optimization of patient-centered care in the outpatient setting.

## Figures and Tables

**Table 1:** Baseline characteristics of patients with APC who received chemotherapy (*n* = 123).

Characteristic	*n* (%)
Median Age at diagnosis	65 (range 42–88)
<65	57 (46.3)
≥65	66 (53.7)
Sex	
Male	65 (52.8)
Female	58 (47.2)
Histology	
Adenocarcinoma	119 (96.7)
Other *	4 (3.3)
ECOG	
0–1	101 (82.1)
2–3	19 (15.4)
Unknown	3 (2.4)
Metastatic disease	
Yes	75 (61.0)
No	48 (39.0)
Tumor Location	
Head/Neck	76 (61.8)
Body	26 (21.1)
Tail	18 (14.6)
Unknown	3 (2.4)
Chemotherapy Regimen	
FOLFIRINOX	85 (69.1)
Nab-Paclitaxel + Gemcitabine	27 (22.0)
Gemcitabine	11 (8.9)
Number of visits with ESAS-r assessment	
1	16 (13.0)
2	12 (9.8)
3	5 (4.1)
4	9 (7.3)
5	7 (5.7)
6–10	23 (18.7)
11–20	23 (18.7)
>21	28 (22.8)
Median Progression Free Survival	6.7 months
Median OS	10.2 months

* Other histologies include: adenosquamous carcinoma, sarcomatoid carcinoma, acinar cell carcinoma.

**Table 2:** Most common moderate-to-severe symptoms at baseline.

Symptom	*n* (%)
Fatigue	70 (56.9)
Anxiety	62 (50.4)
Shortness of breath	60 (48.8)
Nausea	12 (9.8)

**Table 3:** Cox regression predicting survival (baseline measures of TSDS, Physical, and Psychological Scales).

Variable		Univariable			Multivariable		
		HR	95% CI	*p*	HR	95% CI	*p*
TSDS	per 10	1.10	0.99–1.23	0.081	0.99	0.84–1.18	0.951
Physical ≥ 4	Yes	1.33	0.84–2.13	0.228	1.22	0.66–2.25	0.519
	No	1			1		
Psychological ≥ 4	Yes	1.29	0.88–1.90	0.197	1.15	0.69–1.91	0.588
	No	1			1		
Metastasis	Yes				1.74	1.14–2.65	0.010
	No				1		
CT regimen	FOLFIRINOX				0.33	0.17–0.67	0.002
	Nab-Paclitaxel/Gem				0.38	0.17–0.85	0.019
	GEM				1		
Age	(in years)				1.01	0.99–1.03	0.321
ECOG	≥2				1.54	0.81–2.93	0.190
	0–1				1		

**Table 4:** Cox regression from joint model predicting survival (baseline measures of physical and psychological scales plus time-varying TSDS).

Variable		Univariable			Multivariable		
		HR	95% CI	*p*	HR	95% CI	*p*
TSDS (time-varying)	per 10	1.68	1.43–1.97	<0.001	1.65	1.39–1.95	<0.001
Physical ≥ 4	Yes	0.62	0.35–1.07	0.087	0.66	0.37–1.20	0.171
	No	1			1		
Psychological ≥ 4	Yes	0.99	0.64–1.54	0.961	0.91	0.58–1.43	0.689
	No	1			1		
Metastasis	Yes				2.05	1.3–3.22	0.002
	No				1		
Treatment	FOLFIRINOX				0.59	0.29–1.23	0.163
	Nab-Paclitaxel/Gem				0.58	0.25–1.36	0.212
	GEM				1		
Age	(in years)				1.02	0.99–1.04	0.147
ECOG	≥2				1.35	0.71–2.56	0.36
	0–1				1		

## Data Availability

The data presented in this study are available on request from the corresponding author. The data are not publicly available due to ethical/privacy concerns.
